# Phubbing and Social Intelligence: Role-Playing Experiment on Bystander Inaccessibility

**DOI:** 10.3390/ijerph181910035

**Published:** 2021-09-24

**Authors:** Eerik Mantere, Nina Savela, Atte Oksanen

**Affiliations:** 1 Faculty of Social Sciences, Tampere University, 33014 Tampere, Finland; nina.savela@tuni.fi (N.S.); atte.oksanen@tuni.fi (A.O.); 2 Faculty of Sociology, University of Bordeaux, 33000 Bordeaux, France

**Keywords:** smartphones, phubbing, social intelligence, bystander inaccessibility

## Abstract

Smartphone use has changed patterns of online and offline interaction. Phubbing (i.e., looking at one’s phone instead of paying attention to others) is an increasingly recognized phenomenon in offline interaction. We examined whether people who phub are more likely to have lower social intelligence, whether phubbing is considered more annoying than being ignored due to reading a magazine, and if people describe smartphones and magazines differently as sources of social distraction. We collected two survey samples (*N* = 112, *N* = 108) for a cartoon-based role-playing experiment (the Bystander Inaccessibility Experiment) in which a smartphone user and a person reading a magazine ignored the respondents’ conversational initiatives. Annoyance in each scenario was measured, and written accounts were collected on why the respondents rated the scenarios the way they did. Other measures used included the Generic Scale of Phubbing, Generic Scale of Being Phubbed, and Tromsø Social Intelligence Scale. The results showed that participants in both samples were more annoyed by phubbing than by being ignored due to reading a magazine. Linear regression analyses showed that phubbing was associated with lower social intelligence, even after adjusting for confounding factors. The annoyingness of phubbing was explained with negative attitudes toward smartphones, which were assumed to be used for useless endeavors, while magazines were more appreciated and seen as more cultivating. The role of bystanders’ epistemic access to the smartphone user’s activities is discussed.

## 1. Introduction

Smartphone use is booming. In the United States, 96% of people between the ages of 18 and 29 own a smartphone [1]. Smartphones are used regularly during moments together with friends and family. This has caused much debate on smartphone absorption and politeness [2,3]. This article investigates annoyance caused by smartphone use and inattentiveness in social situations; we focused on how and why people find these situations annoying and whether lower social intelligence is associated with ignoring others by smartphone use.

Smartphones have an important quality that sets them apart from other everyday objects: they are used routinely for multiple purposes but give few cues to bystanders about what they are actually being used for. This bystander inaccessibility (BI) makes smartphones uniquely apt in creating socio-cognitive ambiguity on an encounter’s social frame [4]. According to Goffman, frames are fundamental for intersubjectivity and successful interaction in any social situation [5]. People in social situations figuratively ask themselves, “What is it that is going on here?” ([5] p. 7) and use collective understanding of typical types of situations and the types of activities taking place in that particular encounter to find the answer. Interaction derives its meaning from this answer—the local context of interaction, which is renewed continuously through participants’ activities in it. Shared understanding of the social context, including the activities undertaken within it, is necessary for interaction to be intelligible. In classical breaching experiments [6], people reacted with annoyance and anger to the shared understanding being breached without explanation. All major micro-sociological traditions recognize the necessity of shared contextual understanding for interaction [7]. Due to BI, smartphones are likely to create ambiguity over contextually shared understanding. This could be an issue especially for those oriented toward succeeding in social situations, i.e., the “socially intelligent.”

The term “phubbing” was created by a marketing agency for increasing their client’s dictionary sales. It was defined as “*snubbing someone in a social setting by looking at your phone instead of paying attention*”. The campaign imitated organic social media and featured at a “Hall of Shame” on its site, which encouraged site visitors to “*be brutal*” and post photos of their loved ones guilty of phubbing [8]. Snubbing is defined as “*to check, reprove, or rebuke in a sharp or cutting manner; in later use, to treat or receive (a person, suggestion, etc.) in a way calculated to repress or mortify*” [9]. Phubbing has thereafter reached a less drastic meaning, in both common use and modern dictionaries: “*the practice of ignoring one’s companion or companions in order to pay attention to one’s phone or other mobile device*” [10]. Regardless of the term’s origins, researchers have widely adopted phubbing as a term. Though it presumes intent, it is also used in studies in which the phubber’s intent cannot be assumed. This study adopts this now commonplace usage of the term.

Phubbing may be felt as distracting and undermining the benefits of social interactions [11]. It may decrease the quality of communication and relationship satisfaction by lessening the feeling of being together [12], may be negatively perceived by its “victims” as well as those who do it themselves [13], and is starting to be viewed as inevitable in today’s societies [14]. Phubbing one’s romantic partner has been found to lower relationship satisfaction and increase conflicts [15], and it can cause depression in long-term marriages [16]. A validated scale for measuring phubbing has been developed [17]. Phubbing risk has been also analyzed with a conceptual model of communication disturbances and phone obsession [18].

Conceptually, phubbing is close to technoference, defined by McDaniel and Coyne as the “*everyday intrusions or interruptions in couple interactions or time spent together that occur due to technology.*” [19] Technoference has been studied in parent–adolescent relationships [20] and was connected to lower life satisfaction and depression as well as lowered relationship quality between parents and their teenage children, due to conflicts over technology use. Technoference in parent–child interactions was connected to behavior problems among children [21,22] and negative outcomes among adolescents [20].

Interactive research on phubbing is scarce [23]. Licoppe and Figeac studied how smartphone use while driving interacted with traffic light stops. Users timed disengagement from smartphone use to the smartphone interface’s affordances of transition-relevant places, where shifts in orientation between the smartphone and environment were sequentially proffered. The results showed that not all moments of smartphone usage were equally apt for disengaging from its use [24]. Figeac and Chaulet studied smartphone use in public transport and found that gaze shifts away from the phone were organized in relation to the sequential progression of the smartphone activity. They suggested that the beginning stages of smartphone use were especially sequentially engaging [25].

This article answers a frequent call for research on contextual specificities of collocated interaction and mobile digital media use [26]. Furthermore, we aim to understand phubbing in the context of social intelligence. Social intelligence is used to refer to individual differences in understanding others and succeeding in social situations [27]. It has been studied in the contexts of human cognition development within societies [28,29], leadership [30], and reading of nonverbal cues [31]. Academically, the concept has recently overlapped with emotional intelligence, which is sometimes used as a synonym for social intelligence [32]. However, these concepts should be kept separate as emotional intelligence often is used to depict strictly intrapersonal capacities. Although previous research has examined problematic phone use in relation to introspective emotional intelligence [33], social intelligence and phubbing have not yet been studied. Our research questions are as follows:

RQ1. Are people who phub more likely to have lower social intelligence?
RQ2. Is phubbing considered more annoying than ignoring others due to reading a magazine?
RQ3. How do people explain their annoyance with phubbing in relation to being ignored due to reading a magazine?

## 2. Materials and Methods

### 2.1. Participants

The data were obtained from two convenience samples. Data were collected from sample 1 in 2016 (*N* = 112) and from sample 2 in 2018 (*N* = 109) from Finnish university students. The 2016 sample data were collected as a pilot study and exploration of the phenomenon. This sample include Bystander Inaccessibility Experiment only. Sociodemographic information was not collected, but the sample involved university students, the majority of which were women. The 2018 sample participants were predominantly young (*M*_age_ = 26.83, *SD*_age_ = 7.79; *Mdn*_age_ = 23.00) women (86%). A combined dataset was used for the qualitative analyses (*N* = 221). The participants for both studies were recruited from the same first-year social sciences course at a Finnish university. The data collection procedure was the same, and participation was part of completing the course. The survey was conducted in Finnish.

### 2.2. Measures

Quantitative and qualitative data were analyzed in dialog with each other. Quantitative analyses of sample 1 informed its preliminary qualitative analysis, which again informed the measures and analyses of sample 2.

#### 2.2.1. Bystander Inaccessibility Experiment, First Version (BIE-1)

**Figure 1:**
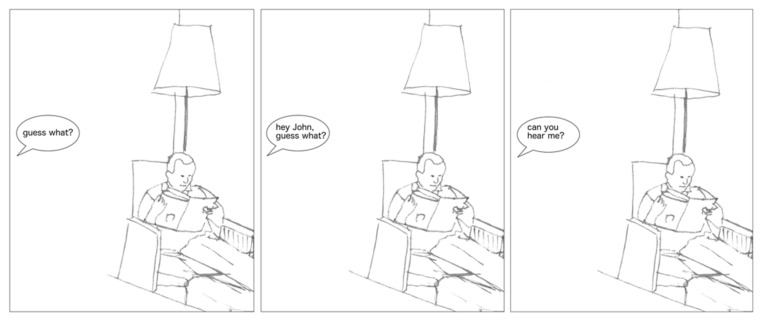
Magazine situation.

**Figure 2:**
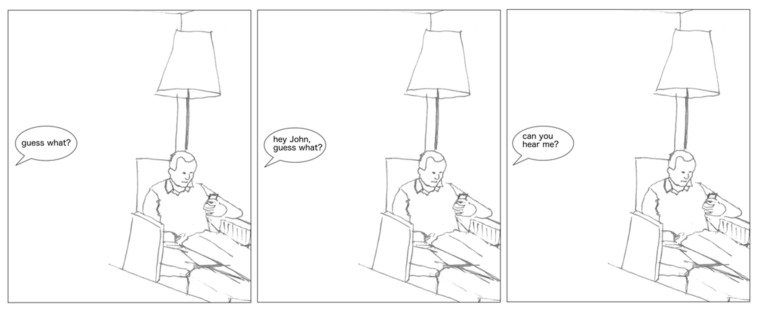
Smartphone situation.

The first version of the Bystander Inaccessibility Experiment was developed to test whether being ignored due to the use of a media artifact instigating less BI sparks less annoyance. Representative episodes of phubbing and being ignored due to another type of activity were developed based on studies using naturalistic data [23]. An anonymized cartoon-based representation was constructed of situations of being ignored due to smartphone use vs. due to reading a magazine (see Figure 1 and Figure 2). The respondents were given the following instructions: *“Put yourself in the POSITION OF THE PERSON SPEAKING and evaluate how annoying the situation would be for you”.* The respondents then rated the situations on a scale from 1 to 7 (1 = not at all annoying, 2 = not very annoying, 3 = a little annoying, 4 = somewhat annoying, 5 = quite annoying, 6 = very much annoying, and 7 = extremely annoying). In the 2016 version, the scale was from 1 to 5 (1 = not at all annoying, 2 = not very annoying, 3 = somewhat annoying, 4 = quite annoying, 5 = very annoying). In both versions, the order of the cartoons was randomized. Two BIE-1 variables were used in the quantitative analyses, hereafter referred to as “Magazine” and “Smartphone.” After evaluating both situations, the respondents were asked, “*Why did you evaluate the first and the second situation as you did?”*

#### 2.2.2. Generic Scale of Phubbing, Finnish Version (GSP-FV)

The Generic Scale of Phubbing is a 15-item questionnaire measuring phubbing behavior [17]. At the start of the questionnaire, respondents are given the following instructions: “*We would like you to think about your mobile phone use during your face-to-face social interactions with others*”. This is then followed by further guidance: “*Think about your social interactions on the whole (e.g., with friends, acquaintances, family, and your partner) and the extent to which the following statements apply to you. In my face-to-face social interactions with others…*” Respondents then rate statements related to their phubbing behavior on a scale from 1 to 7, with labels attached to each number (1 = never, 2 = rarely, 3 = occasionally, 4 = sometimes, 5 = frequently, 6 = usually, and 7 = always). This study is the first to use a Finnish version of the GSP. The measure had good internal consistency, based on McDonald’s omega (ω = 0.88).

#### 2.2.3. Generic Scale of Being Phubbed, Finnish Version (GSBP-FV)

The Generic Scale of Being Phubbed is a 22-item questionnaire measuring the prevalence of being phubbed by one’s social contacts [17]. The scale and the second instruction are identical to those of the GSP, but instead of instructing the respondent to focus on their own mobile phone use, the first instruction is as follows: *“We would like you to think about others’ mobile phone use during your face-to-face social interactions with others”.* This study is the first to use a Finnish version of the GSBP. The measure had an excellent internal consistency (ω = 0.94).

#### 2.2.4. Tromsø Scale of Social Intelligence, Finnish Version (TSIS-FV)

The Tromsø Scale of Social Intelligence is a 21-item questionnaire measuring social intelligence [27]. It has subscales for social information processing, social skills, and social awareness [27]. It was developed for a Norwegian-speaking sample but has been validated and used in English [34], Italian [35], and Korean [36], among other languages. Respondents are provided the following instructions: *“For each item, indicate how well it describes you on a scale from 1 (describes me extremely poorly) to 7 (describes me extremely well)”.* Labels were not provided for values from 2 to 6. This study is the first to use a Finnish version of the TSIS. The measure had excellent internal consistency (ω = 0.90).

#### 2.2.5. Background Variables

Other variables included age, gender, income, and whether the participants had children. These were treated as control variables and used as dummy variables. The age variable indicated if the participant was 23 years old or older, the gender variable indicated female gender, and the income variable indicated a relatively high monthly income for a student (EUR 1200 or over).

### 2.3. Analysis Techniques

The data from samples 1 and 2 were pooled together for qualitative analysis. The datasets underwent quantitative analysis separately.

#### 2.3.1. Quantitative Analysis Methods

We used *t*-tests to analyze sample 1 and report the t-statistics, means (*M*), standard errors (*SE*), standard deviations (*SD*), and confident intervals (*CI*). For the 2018 sample, in addition to the descriptive statistics of our study variables, we report Pearson correlation coefficients and *p*-values from the descriptive analysis and unstandardized (*B*) and standardized (β) regression coefficients, standard errors (SE (B)), and *p*-values for the ordinary least squares (OLS) regression models. Based on qualitative assessment, some 2018 sample participants (*n* = 8) might not have understood the experiment conditions correctly. However, as the results did not change when excluding these participants, our analyses included the entire 2018 sample (*N* = 108).

#### 2.3.2. Qualitative Analysis Methods

Thematization and qualitative content analysis were utilized to categorize the themes among the written responses to the BIE-1 (*N* = 221). The Key Word in Context routine, thesauruses, and NVivo 12 were used. The respondents often referred to several topics, making the number of codes larger than that of total respondents. The themes were defined as reasons for evaluating the smartphone and magazine situations equally (non-differentiators, ND: 355 codes and themes) or differently (differentiators, D: 676 codes and themes) as causes of annoyance. All D codes, except from participants who seemed to have misunderstood the assignment (*n* = 12; e.g., identified with the wrong person), had evaluated the smartphone situation as more annoying. Uninformative verbal repetitions of the numeric evaluation (e.g., *“Both situations, in my mind, were equally annoying”*) were not coded. Elaborations on the annoyance (e.g., “*It is insulting to not answer”*) were coded, even if they did not explicitly mention smartphones and/or magazines.

The aim of the coding was minimal loss of content due to abstraction. Parallel codes were merged, leaving 639 codes. Similar codes were grouped into themes and subthemes. The themes were abstracted into main themes by re-examining and comparing themes, subthemes, codes, and the original written accounts. Several cases clearly referring to a misunderstanding of the experiment (*n* = 12) were grouped together and set aside.

## 3. Results

### 3.1. Quantitative Results

The results of sample 1 from 2016 are shown in Table 1. Based on our results, being phubbed is more annoying than being ignored due to reading a magazine (*t* [106] = −9.10, *p* < 0.001). None of the respondents ranked the magazine condition as more annoying than the smartphone condition.

The results of sample 2 replicated these findings: again, being phubbed was seen as more annoying than being ignored due to reading a magazine (*t* [107] = −5.15, *p* < 0.001). Of the respondents, only 11% (13/108) considered the magazine condition more annoying than the smartphone condition.

Table 2 shows the correlation analysis results for sample 2. We found that social intelligence was correlated negatively with phubbing (*r* = −0.32, *p* < 0.001) and being phubbed (*r* = −0.21, *p* = 0.033) but not with the other study variables. Phubbing was connected positively to being phubbed (*r* = 0.21, *p* = 0.026) and annoyance in the magazine condition (*r* = 0.29, *p* = 0.003), and negatively to age (*r* = −0.27, *p* = 0.005) and income (*r* = −0.20, *p* = 0.040). In addition, being phubbed was linked positively to annoyance in the magazine (*r* = 0.34, *p* < 0.001) and smartphone (*r* = 0.34, *p* < 0.001) conditions, and annoyance in both conditions was correlated with each other (*r* = 0.34, *p* < 0.001). No statistically significant correlations were found between the other variables.

The regression analysis results are shown in Table 3. Based on our analysis, phubbing was a strong negative predictor of social intelligence (β = −0.36, *p* < 0.001). The association between being phubbed and social intelligence was not statistically significant (β = −0.19, *p* = 0.061). Other factors of annoyance in the magazine (β = 0.11, *p* = 0.303) and smartphone (β = 0.05, *p* = 0.650) conditions, i.e., age (β = −0.21, *p* = 0.063), female gender (β = 0.15, *p* = 0.091), whether the participant had children (β = 0.01, *p* = 0.946), and income (β = 0.18, *p* = 0.075) were also not connected to social intelligence.

### 3.2. Qualitative Results

The qualitative analysis resulted in 8 main themes, 47 themes, 35 subthemes, and 639 codes. The accounts of D (676 codes and themes) were more prolific and reflective than those of ND (355 codes and themes). D and ND also differed in the themes to which they referred to (see Table 4).

**Object usage** groups together moral and functional qualities attached to smartphones and magazines. A magazine was considered a better reason for ignoring someone (*n* = 64), by virtue of being more important (*n* = 20), affording self-development (*n* = 5), and being, for instance, civilizing (*n* = 2). A smartphone was deemed a bad reason for ignoring someone (*n* = 79) due to being unimportant (*n* = 58) or useless (*n* = 41) or probably just being used for entertainment and social media (*n* = 10). Only two respondents explicitly addressed the possibility of smartphones being used for something worthwhile. Many struggled to understand why phubbing felt so much worse than being ignored due to reading a magazine (*n* = 63). Some participants (*n* = 2) attributed this to the category and goal of the smartphone user’s actions being inaccessible to the bystander: *“The first situation [smartphone] was really annoying because I didn’t know what was so much more important than what I wanted to say”* (r25_2016). Positive aspects of reading and negative aspects of smartphone use were the most common references in D (*n* = 195). Reflections related to bystander inaccessibility were common in D (*n* = 52) but also appeared in ND (*n* = 11). Some saw relevance in smartphone use being physically more active than reading a magazine (*n*_D_ = 8, *n*_ND_ = 2).

The **intentionality** of ignoring another was a common theme in both D (*n* = 69) and ND (*n* = 44). However, magazine absorption (*n* = 36) and concentrating on reading a magazine (*n* = 24) were always described positively (e.g., *“Smartphone use is a more annoying reason to ignore someone than reading a magazine. The latter is actually more amusing because it’s admirable if someone can focus on reading that much. On the other hand, scanning your phone seems like some technical apparatus is more important than I am”* (r19_2018). Absorption and concentration were never mentioned positively in relation to smartphones. Hearing the questions but choosing to ignore them was used to explain annoyance in 18 cases. Assumed inability to hear was seen as decreasing annoyance (*n* = 4), except in one case, in which not hearing due to smartphone use actually increased the participant’s annoyance: “*I think in the first [smartphone] situation, the person doesn’t actually even hear me. My assumption is based on previous experience, which numbers in the hundreds. It’s like talking to a tree when someone’s on their phone. I think he’s more out of reach when he’s on his phone than when he’s with a magazine”* (r20_2018). Smartphones were said to not actually demand attention as reading a magazine does. Smartphones were also associated with addiction, said to destroy one’s ability to focus, used for escaping negative emotions, and be destructive to brains. Absorption in reading a magazine was described positively, even as *“endearing”* or manifesting a *“joy of living.”* Smartphone absorption was never described positively.

**Societal factors** (*n* = 78) were used to explain annoyance in D through the overly high prevalence of smartphone use (*n*_D_ = 36, *n*_ND_ = 4). Phubbing was seen to take place too often (*n* = 30), creating a contrast between the world today and the world before, when people still paid more attention to one another (*n* = 19). Five respondents talked about the prevalence of smartphone addiction in society. Smartphone use was described as having gone too far and taking too big a portion of peoples’ lives. Exceptions to this included the accounts in which both situations were framed as normal everyday life (*n*_ND_ = 3) as well as comments on the lack of basic manners and morality causing equal annoyance in both situations (*n*_ND_ = 6). Although smartphone use was often described in many negative moralistic terms, those judgmental accounts never referred similarly to potential social norms.

**Interpersonal relations** groups together references to being less than an imagined other, references to the social situation’s constitution, and reflections on oneself as an interlocutor. Other potential people on the receiving end of the smartphone usage were seen as competitors for the bystander’s attention, who were perceived as being more important than the bystander and made the bystander feel as though they were a *“third wheel”* in the situation (*n*_D_ = 19, *n*_ND_ = 2). Yet, the presence of the smartphone-mediated others was considered somehow inconcrete and as a less urgent and less valuable form of social interaction when given the option for offline interaction: *”Perhaps it also makes you feel a bit like the contents of the phone and the other people there are more interesting than concrete human company”* (r43_2016).

**Non-responsiveness** was described as being insulting in general (*n* = 28). The category had overlap with the theme of intentionality. When non-responsiveness was described in relation to smartphones, the respondents often assumed intention. This was contradictory as the data also included rich accounts of the ease of losing awareness of one’s surroundings due to smartphone use. Smartphone users’ non-responsiveness often was assumed to take place after hearing the other person but choosing to ignore them. Culpability sometimes was attributed to the phubber even when the phubber was thought to not hear the question. The rationale was that, in choosing to engage with their phone to the degree that they lose awareness of their surroundings, the phubber has already decided intentionally to neglect others’ possible conversational initiatives. Some accounts and references to the general annoyingness of having to repeat oneself (*n* = 15) or waiting for a reply (*n* = 15) formed the largest themes explaining annoyance among ND.

**Presence** was considered lacking due to smartphone use (*n*_D_ = 11, *n*_ND_ = 1): “When someone’s absorbed in their smartphone, it feels as though they’re in ‘another world’ even if they’re in the same physical space” (P16_32), although some instances of ND referred to absence in general as annoying (*n*_ND_ = 4): “That also adds to the annoyance—that John is not present with me in the same physical situation but his attention is elsewhere” (r47_2018). References to presence overlapped with imagining the phubber being engaged with others: “In the first [smartphone] situation, the person was possibly interacting with some other person via the phone and wasn’t present in the situation.” (r4_2018).

**Incited emotions** included elaborations on the emerging emotions attributed to the situations. Both the D (*n* = 12) and ND (*n* = 8) responses made references in this category. For D, the most common emotional theme was the insulting nature of phubbing (*n*_D_ = 5, *n*_ND_ = 1); for ND, it was the annoyance over the listener’s perceived indifference (*n*_D_ = 1, *n*_ND_ = 3). The respondents felt unimportant in general, and some felt even more unimportant specifically in the smartphone situation. Phubbing was called *“enraging”* and *“simply annoying”.* Similarly drastic and laconic descriptions were not given for being ignored by another due to magazines.

## 4. Discussion

We investigated annoyance caused by smartphone use and inattentiveness in social situations, specifically phubbing. The study hypotheses were based on the ethnomethodological theory of social action, which posits that the context of social behavior indexically defines its meaning, and the behavior in the context reflexively defines the context [6]. Therefore, smartphones—which may be used for more varied purposes than printed media such as magazines can, and typically keep this purpose inaccessible to bystanders [4]—would stand out as a special source of distraction in social settings. We found phubbing to be connected to lower social intelligence. Our two samples also showed that being phubbed was considered more annoying than being ignored due to reading a magazine, and that this was typically caused by people’s perception of smartphones as being a worse reason for ignoring others than reading a magazine was.

Reading a magazine was seen positively, while smartphone use was seen negatively. Magazines were described as educational, civilizing, and good for developing one’s concentration. Smartphones were thought to destroy one’s ability to concentrate. Being absorbed in reading a magazine was considered more understandable and even was described as admirable, sometimes even if it led to being ignored by the absorbed reader. Absorption in one’s smartphone was never depicted positively and was even described as “enraging.” One respondent would have been more annoyed with the phubber, even if she knew he was reading the same exact thing on his smartphone, than with the reader in the magazine condition. This reflects the findings of previous studies showing negative bystander reactions to smartphone absorption already in childhood [37].

The assumed negative impact of smartphone use on character development, and the positive impact of reading a magazine, directly influenced respondents’ perceived annoyance. High relevance of the activity attributed as the cause of being ignored makes sense, from an ethnomethodological point of view. The meaning of smartphone use or magazine reading indexically gives meaning to being ignored in the situation. Due to BI, being ignored in the smartphone situation had more ambiguous meaning and significance. This may partly explain why accounts of annoyance over phubbing were so much more extensive, diverse, and explorative than those relating to magazine reading. Without understanding the nature and goal of the activity causing one to be ignored, the sense of being ignored remains undefined. In their written accounts, respondents therefore made great efforts in searching for ways to define that nature and goal.

Some respondents addressed the ambiguity of phubbing directly. They remarked that unlike in the magazine condition, where they knew the person was reading, in the smartphone condition, they had no idea what the other person was doing specifically, but few of the accounts addressed this lack of epistemic access into smartphone activities. However, interaction generally is organized through routinized moral orientations toward shared norms of social cooperation [6]. People treat themselves and each other as accountable for comporting themselves in such a manner that the sense of their actions in a social situation is readily deductible from their observable conduct and the setting’s attributes [6]. This norm of accountability is typically sanctioned rapidly if not met, but due to its routinized nature, its guiding impact on our social lives is rarely addressed or reflected upon explicitly [6]. This raises the possibility that BI, i.e., the lack of bystander epistemic access into a smartphone user’s activities, may affect experiences of being phubbed when not explicitly addressed. Many respondents’ bewilderment over the difference they felt between the smartphone and magazine conditions supports this line of thinking.

While manners and morality were addressed in a laconic manner in relation to the general norms of ignoring another person, the wrongness of phubbing was addressed together with elaboration and justifications. This would suggest that, although phubbing is a commonly disliked phenomenon, unequivocal social norms have not yet been formed to regulate it. This is understandable as social norms become naturalized through transgenerational transmission [38]. However, it may be impossible to predict whether these norms will form in the future. The relevant technologies develop faster than generations change, thus not allowing the transgenerational transmission of social norms for these technologies. Considering mobile digital media’s pivotal role in today’s societies, this is an enormous topic, which multidisciplinary scientific communities should study. If the general norms of accountability in social behavior were to change to accommodate phubbing behavior, this would change what it means to be successful in social situations—that is, what it means to be socially intelligent.

### Limitations and Future Directions

The study used Finnish translations of the GSP and GSBP and a translation of an English version of the TSIS. Although all three scales showed good construct validity, this study was limited to Finnish university students. Though gender did not have significant impact on results of statistical models, the study is limited by the qualitative results predominantly reflecting female reactions to phubbing. Future studies should use internationally representative samples and aim to confirm the minor evidence we found on the connections between social intelligence, age, and being phubbed using different study designs and methods. The BIE-1 should be elaborated upon and developed to better extract the role that BI plays in reported feelings of annoyance.

The term “phubbing” is limited by dichotomous views on attention. In social situations with phubbing, the allocation of one’s attention to engagement in one’s smartphone and co-present others is not either-or; rather, it manifests in degrees through the allocation of interactive resources such as one’s words, gaze, and corporal configurations, in relation to the device and collocated others [4]. Instead, phubbing should be recognized as a moralistic term, depicting the attitude of the user of the term on the balance of face-to-face and smartphone engagements embodied by another.

The study supports a direction for human–computer interaction research with its focus on activities. Activities and epistemic access to them by others may be more socially relevant than the platforms and applications used. Non-digital media devices are typically self-defined by the classes of activities they afford. Further explanations of their use would typically be redundant. When a person has a course textbook in their hands, he or she clearly is assumed to be studying and not looking at their favorite influencer’s Instagram photos. This epistemic access to user activities typically is lacking for mobile digital multipurpose devices such as smartphones. With a smartphone in hand, one might be studying or looking at Instagram photos.

## 5. Conclusions

Smartphones are ubiquitous in today’s everyday face-to-face interactions. Phubbing is an increasingly recognized phenomenon that is potentially disturbing for social situations. Our study findings underline that phubbing not only was seen as a very annoying and negative phenomenon, but was associated with lower social intelligence. The qualitative results showed that the phubber’s smartphone activities were assumed to be unimportant, to absorb the phubber’s attention while not actually requiring it as reading a magazine would, and generally to take too much of people’s time. The intergenerational transmission of social norms around phubbing might be too slow to keep up with the technological development. This might mean drastic changes for how socially intelligent behavior is defined and how social life is organized in general, if social norms adapt to accompany a generally disliked and socially influential behavior such as phubbing.

## Figures and Tables

**Table 1:** Paired sample *t*-test in sample 1.

Variable	*n*	Range	*M*	*SD*	(95% CI)	
Annoyance: Magazine Annoyance: Smartphone	107	1–5	3.13	0.93	2.95	3.31
	107	1–5	3.87	0.87	3.70	4.04
Difference	107		−0.74	0.84	−0.90	−0.58

**Table 2:** Correlations and descriptive statistics for the main variables in sample 2.

**Continuous variables**	**Range**	***M* (*SD*)**	**1**	**2**	**3**	**4**	**5**	**6**	**7**	**8**
1. Social intelligence	1–7	5.13 (0.86)								
2. Phubbing	1–7	2.49 (0.73)	−0.32 ***							
3. Being phubbed	1–7	3.42 (0.87)	−0.21 *	0.21 *						
4. Annoyance: Magazine	1–7	4.45 (1.35)	−0.02	0.29 **	0.34 ***					
5. Annoyance: Smartphone	1–7	5.12 (1.44)	−0.01	0.19	0.34 ***	0.54 ***				
**Categorical variables**	**Range**	**%**								
6. Age > 23 y.o.	0/1	46.79	−0.04	−0.27 **	−0.09	−0.05	−0.09			
7. Female gender	0/1	86.24	0.13	0.10	−0.03	0.03	−0.01	0.03		
8. Has children	0/1	22.94	−0.01	−0.13	0.09	0.04	0.05	0.55 ***	0.08	
9. Income ≥ EUR 1200	0/1	27.52	0.19	−0.20 *	−0.07	−0.06	−0.02	0.36 ***	0.05	0.31 **

Note. * *p* < 0.05; ** *p* < 0.01; and *** *p* < 0.001.

**Table 3:** Factors predicting social intelligence.

TSIS	B	*SE* (B)	*p*	β
Phubbing	−0.42	0.12	0.000	−0.36
Being phubbed	−0.19	0.10	0.061	−0.19
Annoyance: Magazine	0.07	0.07	0.303	0.11
Annoyance: Smartphone	0.03	0.06	0.650	0.05
Age > 23 y.o.	−0.36	0.19	0.063	−0.21
Female gender	0.39	0.23	0.091	0.15
Has children	0.02	0.22	0.946	0.01
Income ≥ EUR 1200	0.34	0.19	0.075	0.18

**Table 4:** Main themes, themes, and data extracts.

Object Usage (195/30)	Intentionality (69/44)	Societal Factors (51/27)	Interpersonal Relations (44/20)	Non-Responsiveness (16/20)	Presence (12/9)	Incited Emotions (12/8)
Goodness of replacement activity (133/15), bystander inaccessibility (52/11), corporal behavior (8/2), objects hindering interaction (1/2), magazine not as significant a competitor in getting attention (1/-)	Concentration (17/19), absorption (31/4), hearing or not hearing (5/14), ease of suspending the activity (10/4)	Prevalence in society (36/4), everyday life (3/14), these days (9/3), basic manners and morality (-/6), technology (3/-)	Respondents’ self-reflection (20/14), phubber may be engaged with others (19/2), form of and participants in the social situation (2/4), reader not engaged with someone else (3/-)	Repeating yourself (8/7), lack of reaction is displeasing (4/10), non-response from reader less bad (4/-), perhaps just didn’t hear yet (-/1), waiting is OK because probably reading from a magazine or phone (-/1)	Phones make people absent (11/1), absence is annoying in general (-/4), I require attention when I want it (-/2), being together means paying attention (-/1), inability to create contact (-/1), phubber ignoring me even though I am actually present (1/-)	Phubbing is insulting (4/-), feeling unvalued (2/2), indifference is annoying (1/3), phubbing is frustrating (2/-), phubber makes me feel unimportant (1/1), phubbing is enraging (1/-), not being listened to is frustrating (-/1), both are awkward (-/1), smartphones simply are just annoying (1/-)
*”In the first [smartphone] situation, one doesn’t think that the person is doing anything important. They’re probably just bored, and that’s why they’re staring at their phone”* (r23_2018).	*”A magazine or book is not as annoying because, when reading, people clearly need to concentrate, unlike, for instance, when checking their social media accounts”* (r36_2016).	*”I felt the smartphone was more annoying because people always seem to be on their phones and forget to communicate with the people around them”* (r21_2016).	*”Maybe it is because the person on their phone might be chatting with someone else, and when they do not respond, it feels like the other person is more important than I am”* (r15_2016).	*“The most annoying thing is if you ask if they heard you, and they still don’t answer or start to listen”* (r55_2016).	*“When absorbed in a smartphone, the person almost seems to be in another world, even though I am sharing the same physical space with them”* (r32_2016).	*“I think it is very impolite and insulting not to answer a question because it gives the impression that the other person is not worth your attention or is insignificant”* (r11_2016).

Note. The number of references is given in brackets, for differentiators on the left side of the slash and for non-differentiators on the right side (D/ND). Typical data extracts are presented from each main theme.

## Data Availability

Data will be available from the corresponding author upon reasonable request.
